# Epigenetic inactivation of follistatin-like 1 mediates tumor immune evasion in nasopharyngeal carcinoma

**DOI:** 10.18632/oncotarget.7654

**Published:** 2016-02-24

**Authors:** Xiaoying Zhou, Xue Xiao, Tingting Huang, Chunping Du, Shumin Wang, Yingxi Mo, Ning Ma, Mariko Murata, Bo Li, Wensheng Wen, Guangwu Huang, Xianjie Zeng, Zhe Zhang

**Affiliations:** ^1^ Department of Otolaryngology-Head & Neck Surgery, First Affiliated Hospital of Guangxi Medical University, Nanning, China; ^2^ Medical Research Center, Guangxi Medical University, Nanning, China; ^3^ Department of Environmental and Molecular Medicine, Mie University Graduate School of Medicine, Mie, Japan; ^4^ Faculty of Nursing Science, Suzuka University of Medical Science, Suzuka, Japan; ^5^ Department of Head and Neck Surgery, Affiliated Cancer Hospital of Guangxi Medical University, Nanning, China

**Keywords:** epigenetic inactivation, follistatin-like 1, tumor immune evasion, nasopharyngeal carcinoma

## Abstract

Follistatin like-1 (FSTL1) is a secreted glycoprotein involved in a series of physiological and pathological processes. However, its contribution to the development of cancer, especially the pathogenesis of nasopharyngeal carcinoma (NPC), remains to be elucidated. We aimed to investigate the dysregulation of FSTL1 and its possible function in NPC. *FSTL1* was frequently downregulated in NPC cell lines and primary tumor biopsies by promoter hypermethylation. Ectopic expression of *FSTL1* significantly suppressed the colony formation, proliferation, migration and invasion ability of NPC cells and induced cell apoptosis. Overexpression of *FSTL1* decreased the tumorigenicity of NPC cells *in vivo*. In addition, the proliferation of NPC cells *in vitro* was inhibited by treatment with soluble recombinant FSTL1 protein. The protein level of FSTL1 was decreased in primary NPC tumors and was associated with downregulated interleukin 1β (IL-1β) and tumor necrosis factor α (TNF-α). Furthermore, recombinant human FSTL1 protein induced secretion of IL-1β and TNF-α in macrophage cultures, therefore FSTL1 might activate macrophages and attenuate the immune evasion of NPC cells. In conclusion, the epigenetic downregulation of *FSTL1* may suppress the proliferation and migration of NPC cells, leading to dysfunctional innate responses in surrounding macrophages.

## INTRODUCTION

Nasopharyngeal carcinoma (NPC) is a unique head and neck malignancy with a specific geographical distribution, whose etiology involves a complex cross-interaction between genetic susceptibility, environmental carcinogens and Epstein-Barr virus (EBV) infection [1]. Epigenetic alterations, including aberrant DNA methylation and histone modifications, have been considered important factors in the carcinogenesis of NPC, leading to the silencing of tumor suppressor genes and disruption of important signaling pathways such as Ras and Rho GTPase signaling, cell adhesion and apoptosis signaling [2].

NPC is strongly associated with EBV. EBV DNA has been identified in most undifferentiated NPC. A limited set of potentially immunogenic latency-associated viral antigens is also expressed in NPC cells, which do not evoke antitumor immune responses in the large infiltrate of stromal immune cells in tumors and surrounding tissues. These stromal immune cells might be rendered anergic by factors released in the tumor microenvironment [3, 4].

Follistatin like 1 (FSTL1), also known as FRP or TSC-36, is a secreted glycoprotein and belongs to the BM-40/SPARC/osteonectin family. It was first identified in mouse osteoblastic MC3T3E1 cells, being up-regulated upon TGF-β1 stimulation [5]. *FSTL1* was found downregulated in v-myc and v-ras oncogene-transformed cells, which suggested its possible role in carcinogenesis [6]. It is highly expressed in normal placenta, smooth muscle and many other tissues and cell types and functions in various biological processes, such as cell proliferation, migration, embryonic development, revascularization and cardioprotection [7–10]. Its expression is variable in different malignancies. Overexpression of *FSTL1* was associated with poor prognosis of glioblastoma [11] and favored the progression of prostate cancer [12]; its inactivation in colon, stomach, breast, kidney, lung, endometrial and ovarian cancers suggests a function as a putative tumor suppressor gene [5, 13–15].

To identify novel tumor suppressor genes in NPC, we performed a genome-wide cDNA microarray screening involving treatment with pharmacological demethylating agents. *FSTL1* was 11-fold upregulated with demethylation treatment (unpublished data). Recognized as a pro-inflammatory molecule and an autoantigen associated with rheumatoid arthritis, FSTL1 secretion results in upregulation of interleukin 1β (IL-1β), tumor necrosis factor α (TNF-α), and IL-6 in macrophages and fibroblasts [16, 17].

We hypothesized that promoter hypermethylation in NPC cells might inhibit the paracrine effects of FSTL1 secretion and thus block the cancer–stromal interaction, thereby allowing NPC cells to escape an attack of stromal immune cells. In this study, we first investigated the expression and methylation status of *FSTL1* in NPC cell lines and primary tumor tissues. We then sought to validate the role of *FSTL1* as a tumor suppressor gene by *in vitro* and *in vivo* studies. We also studied the effect of *FSTL1* in regulating macrophages and the immune evasion of NPC cells. Our data suggest the role of *FSTL1* as a tumor suppressor gene in NPC. Epigenetic downregulation of *FSTL1* might lead to the proliferation and migration of cancer cells and inhibit the function of surrounding macrophages.

## RESULTS

### *FSTL1* is downregulated in NPC cell lines and primary tumors

**Figure 1:**
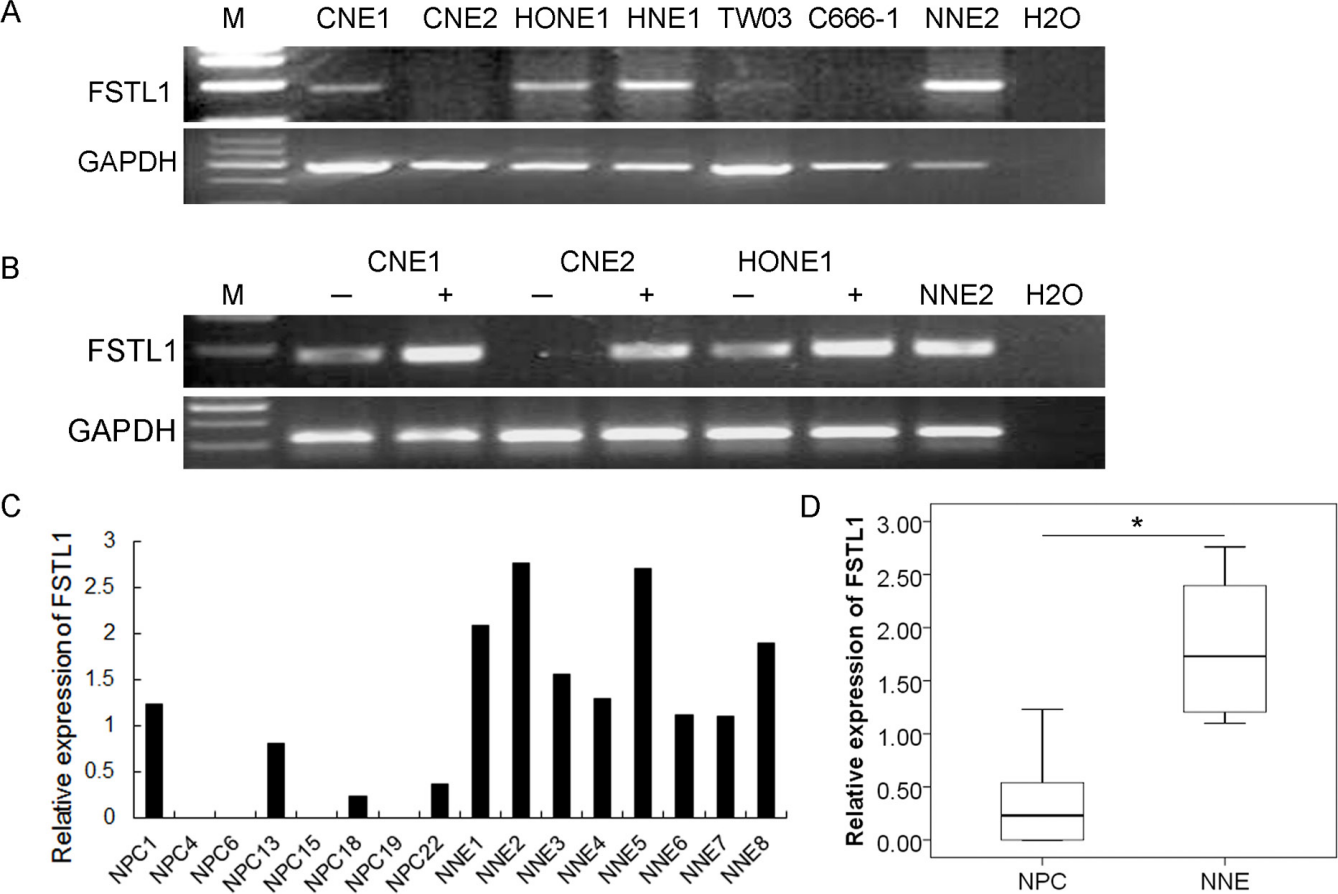
*FSTL1* is downregulated in nasopharyngeal carcinoma (NPC) cell lines and primary human tumor biopsies (**A**) Expression of *FSTL1* in NPC cell lines and normal nasopharyngeal epithelium sample (NNE2). (**B**) Expression of *FSTL1* in three NPC cell lines after treatment with 5-aza-dC. *GAPDH* was an internal control, NNE2 was a positive control. Water was a blank control. (**C**) Semi-quantitative RT-PCR analysis of *FSTL1* mRNA expression in represented primary NPC tumors and NNE tissues (*n* = 8 each). (**D**) Semi-quantitative RT-PCR analysis of *FSTL1* transcription in a total of 25 primary NPC biopsies and 8 NNE samples. The box plots show the ratio of the intensity of *FSTL1* to *GAPDH*. Horizontal line is the mean, box edges are 25 to 75 percentile, and whiskers are 10 and 90 percentiles. *P < 0.05.

We evaluated the transcriptional level of *FSTL1* in NPC cell lines and primary tumors. *FSTL1* expression was significantly inactivated in CNE2 and C666-1 cells and its level was decreased in the NPC cell lines CNE1, HONE1, HNE1 and TW03 (Figure 1A). The demethylating agent 5-Aza-2′-deoxycytidine (5-aza-dC) restored the expression of *FSTL1* in CNE1, CNE2 and HONE1 cells (Figure 1B), which agreed with our cDNA microarray findings (unpublished data). *FSTL1* was downregulated in the 25 NPC primary tumor samples but easily detected in all 8 normal nasopharyngeal epithelium (NNE) samples (*P* < 0.05, Figure 1C, 1D).

### The promoter of *FSTL1* is frequently hypermethylated in NPC cell lines and primary tumors

**Figure 2:**
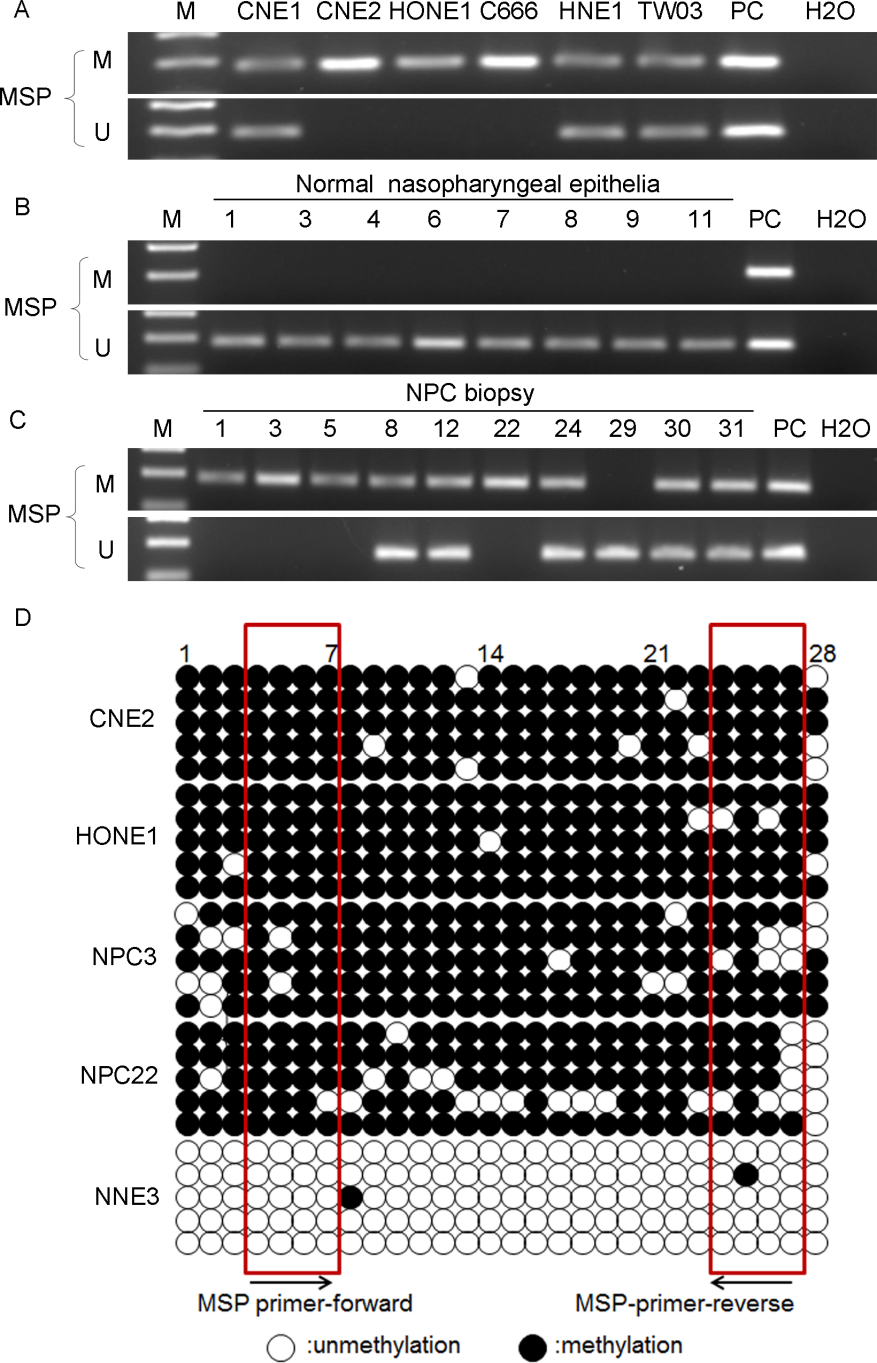
*FSTL1* is aberrantly hypermethylated in NPC cell lines and primary tumors (**A–C**) Methylation-specific PCR analysis of the *FSTL1* promoter region in NPC cell lines and NPC and NNE tissue (representative data are shown). *In vitro*-methylated DNA was a positive control for methylated alleles and DNA from normal lymphocytes was a positive control for unmethylated alleles. The blank control was water. M: methylated alleles; U: unmethylated alleles. (**D**) Bisulfite genomic sequencing of the 28 CpG sites within the promoter region of *FSTL1* in two NPC cell lines (CNE2 and HONE1), two NPC biopsies (NPC3 and NPC22) and one NNE samples (NNE3). Five clones were randomly selected and sequenced for each sample. Each row represents an individual promoter allele analyzed. Open circles are unmethylated cytosines, and filled circles are methylated cytosines. CpG sites covered by MSP primers are shown by frames.

The proximal promoter region of *FSTL1* contains a CpG island that extends into its first exon (−166 bp to +332 bp, data not shown). To identify the methylation status of the *FSTL1* promoter, we performed methylation-specific PCR (MSP) in 6 NPC cell lines (CNE1, CNE2, HONE1, C666-1, HNE1 and TW03), 35 NPC biopsies and 12 NNE samples. The promoter of *FSTL1* was methylated in the 6 NPC cell lines and 68.6% (24/35) of primary NPC samples but no NNE samples (Figure 2A–2C). Unmethylated amplicons were detected in some of the primary NPC tissues but were most likely due to contamination of non-malignant cells such as stromal cells when sampling.

To further reveal the detailed methylation status, we analyzed the 28 individual CpG sites from −288 to −47 bp in the promoter of *FSTL1* by using bisulfite genomic sequencing (BGS). All 28 CpG sites were intensely methylated in CNE2 and HONE1 cells and in 2 NPC biopsies (NPC samples 3 and 22). However, only rare methylated CpG sites were found in the NNE3 sample (Figure 2D). These BGS data suggest complete bisulfite conversion of genomic DNA and further support our MSP results.

*FSTL1* mRNA expression could be restored after treatment with 5-aza-dC (Figure 1B), which suggests that promoter hypermethylation might contribute to the inactivation of *FSTL1* in NPC cells.

### Clinicopathological significance of *FSTL1* promoter hypermethylation in NPC

We examined the association of clinic-pathological parameters of patients and *FSTL1* promoter hypermethylation (Table 1) and found no difference between *FSTL1*-hypermethylated and -nonmethylated cases in patient age, gender, cancer stage, histological subtype and lymph node metastasis, indicating that the promoter hypermethylation of *FSTL1* might be an early event during NPC tumorigenesis.

**Table 1:** Association of clinicopathological characteristics of patients with nasopharyngeal carcinoma (NPC) and follistatin like-1 (FSTL1) promoter methylation status

	No. of patients	*FSTL1* promoter methylation status		*P* value^a^
		Methylated	Unmethylated	
Age, years				NS
< 60	29	19	10	
≥ 60	6	5	1	
Sex				NS
Male	24	16	8	
Female	11	8	3	
Cancer stage^b^				NS
I	4	2	2	
II	10	8	2	
III	10	6	4	
IV	11	8	3	
Histological subtype				NS
Keratinizing squamous cell carcinoma	4	2	2	
Non-keratinizing carcinoma	31	22	9	
Lymph node metastasis				NS
Presence	23	16	7	
Absence	12	8	4	

1. Data are number of patients.

a 2. by Pearson chi-square test or Fisher's exact test.

b 3. according to the International Union Against Cancer (UICC).

4. NS: not significant.

### Overexpression of *FSTL1* suppressed colony formation, proliferation, migration and invasion ability of NPC cells and induced cell apoptosis *in vitro*

**Figure 3:**
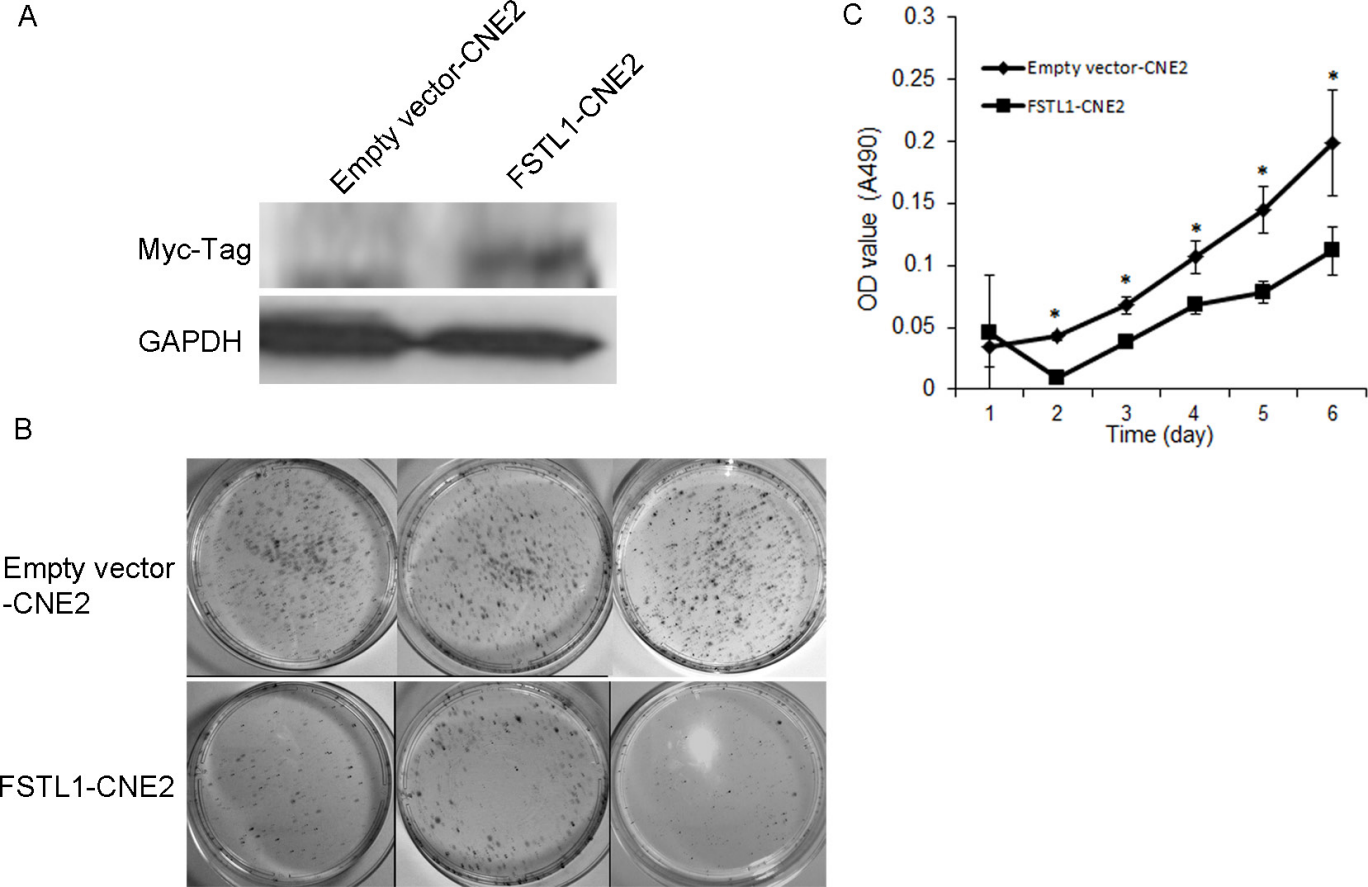
Expression of *FSTL1* inhibits the colony formation and proliferation ability of CNE2 cells (**A**) Western blot assay detecting a fusion protein of FSTL1-Myc tag in stably transfected CNE2 cells. (**B**) Colony formation assay of CNE2 cells transfected in triplicate with *FSTL1*-expressing vector or empty vector and grown for 2 weeks in complete medium containing G418. (**C**) MTT assay of proliferation ability of *FSTL1*-transfected and empty-vector–transfected CNE2 cells. Data are mean ± SD (*n* = 5). *P < 0.05.

To assess the potential tumor suppressor properties of *FSTL1*, we established stable *FSTL1*-expressing CNE2 cells (Figure 3A) and examined their effect on cell colonogenicity, proliferation, migration, invasion and apoptosis. The number and size of colonies was significantly lower in *FSTL1*-CNE2 than empty vector-CNE2 cells (Figure 3B). As well, *FSTL1* re-expression could inhibit NPC cell proliferation (*P* < 0.05, Figure 3C).

**Figure 4:**
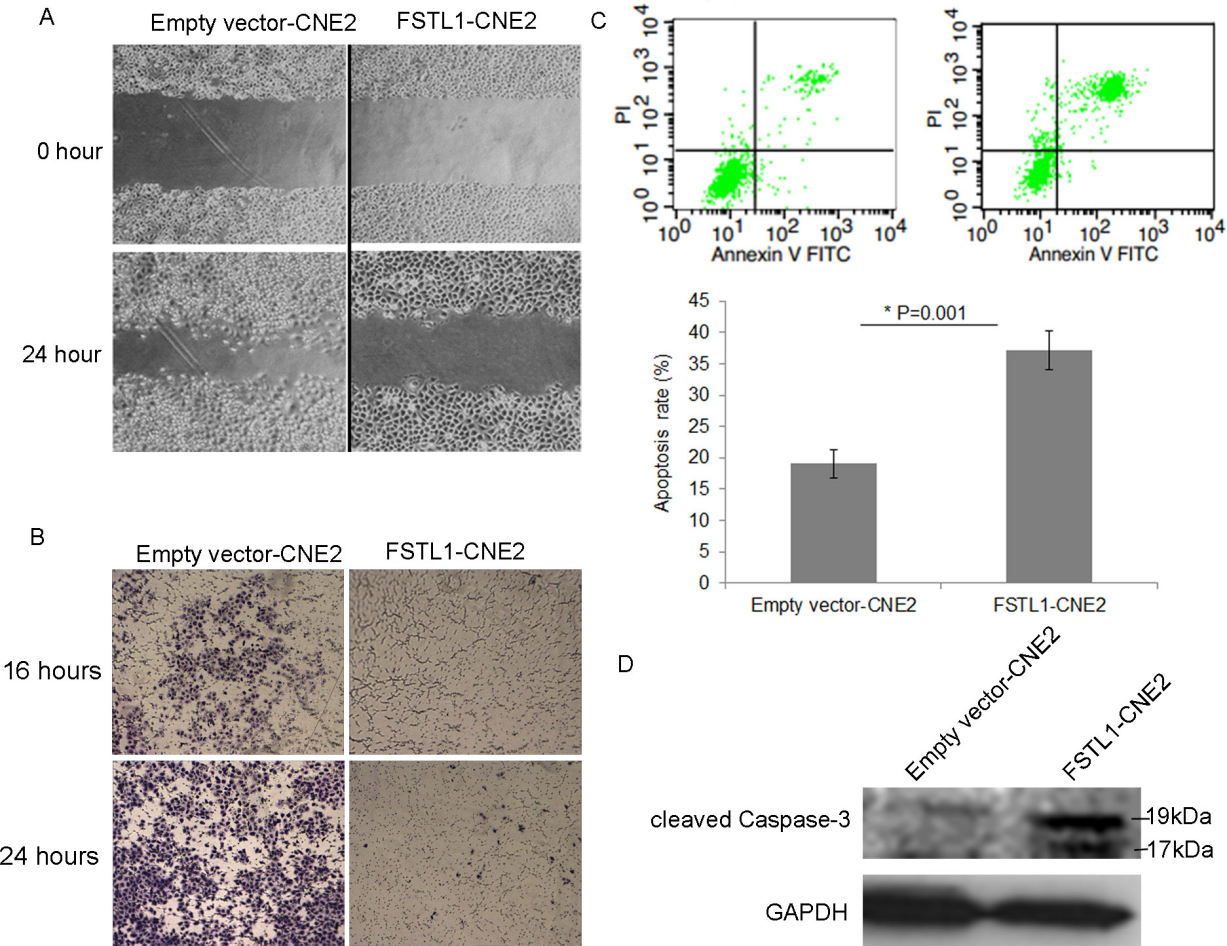
*FSTL1* impedes cell migration, invasion and induces apoptosis of CNE2 cells (**A**) Wound healing assay of the migration of CNE2 cells in response to *FSTL1*. Pictures were taken at the time 0 and 24 h under microscope. Magnification: 40×. (**B**) Transwell assay of the effect of *FSTL1* on invasion capacity of CNE2 cells at 16 and 24 h. Magnification, 40×. (**C**) Flow cytometry analysis of the effect of *FSTL1* on apoptosis and quantification. Cells positively stained with Annexin V-FITC alone (early apoptosis) or Annexin V-FITC and PI (late apoptosis) were considered apoptotic cells. Data are mean ± SD, *n* = 3. (**D**) Western blot assay of cleaved caspase-3 protein level in *FSTL1*-transfected CNE2 cells. GAPDH was an internal control.

To evaluate the impact of *FSTL1* on the motility of NPC cells, we used wound-healing assay. The gap between *FSTL1*-CNE2 cells closed slower than in the control, which suggests that *FSTL1* retards the migration of NPC cells (Figure 4A). We further assessed the effect of *FSTL1* on the invasive capacity of NPC cells by transwell assay. Almost no *FSTL1*-CNE2 cells had invaded 16 h after seeding and only a few cells were observed after 24 h (Figure 4B). In contrast, emptyvector-CNE2 cells showed gradually increased invasion.

In addition, FACS analysis showed that apoptosis rate was greater in *FSTL1*-CNE2 than empty vector-CNE2 cells (37.13 ± 3.13% vs 19.05 ± 2.18%, Figure 4C). Furthermore, protein levels of endogenous cleaved caspase-3 (17 and 19 kDa) were increased in *FSTL1*-CNE2 cells (Figure 4D), which was consistent with FACS results. Therefore, *FSTL1* suppressed the tumorigenic properties of NPC cells *in vitro*.

### Overexpression of *FSTL1* suppressed tumorigenesis *in vivo*

**Figure 5:**
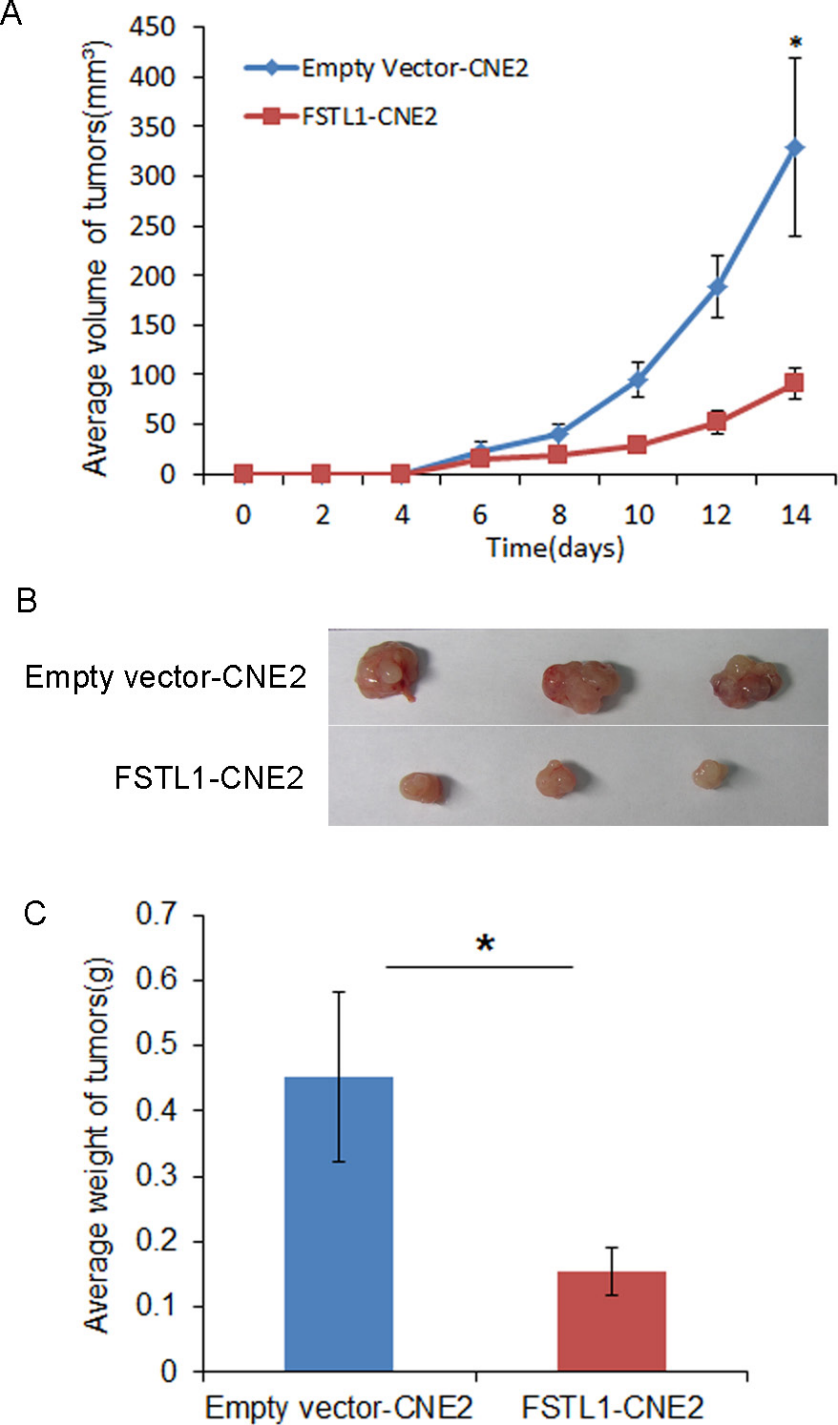
Exogenously expressed *FSTL1* suppresses the tumorigenecity of CNE2 *in vivo* Balb/c athymic nude mice were inoculated with 2 × 10^6^ cells. (**A**) Growth curve of tumors in nude mice. Tumor volume was monitored every 2 days after inoculation. Data are mean ± SD (*n* = 8 for each group). *P < 0.05. (**B**) Tumors were removed from nude mice at day 14 after inoculation; representative data are shown. (**C**) Weight of tumors. Data are mean ± SD (*n* = 8). *P < 0.05.

Stable transfected *FSTL1*-CNE2 and empty vector-CNE2 cells were injected into the right or left flanks, respectively, of nude mice. A 100% tumor formation rate suggested that *FSTL1* does not affect tumor incidence *in vivo*. However, the tumor growth rate was slower in mice with *FSTL1*-CNE2 than control cells (*P* < 0.05, Figure 5A). Fourteen days later, xenografts were removed from mice (Figure 5B), the mean weight of tumors was lower from mice with *FSTL1*-CNE2 than control cells [(0.155 ± 0.037 vs 0.378 ± 0.212 g); *P* < 0.05, Figure 5C]. Thus, re-expression of *FSTL1* in NPC cells may counteract their tumorigenicity *in vivo*.

### Exposure to recombinant FSTL1 protein inhibited the proliferation of NPC cells *in vitro*

**Figure 6:**
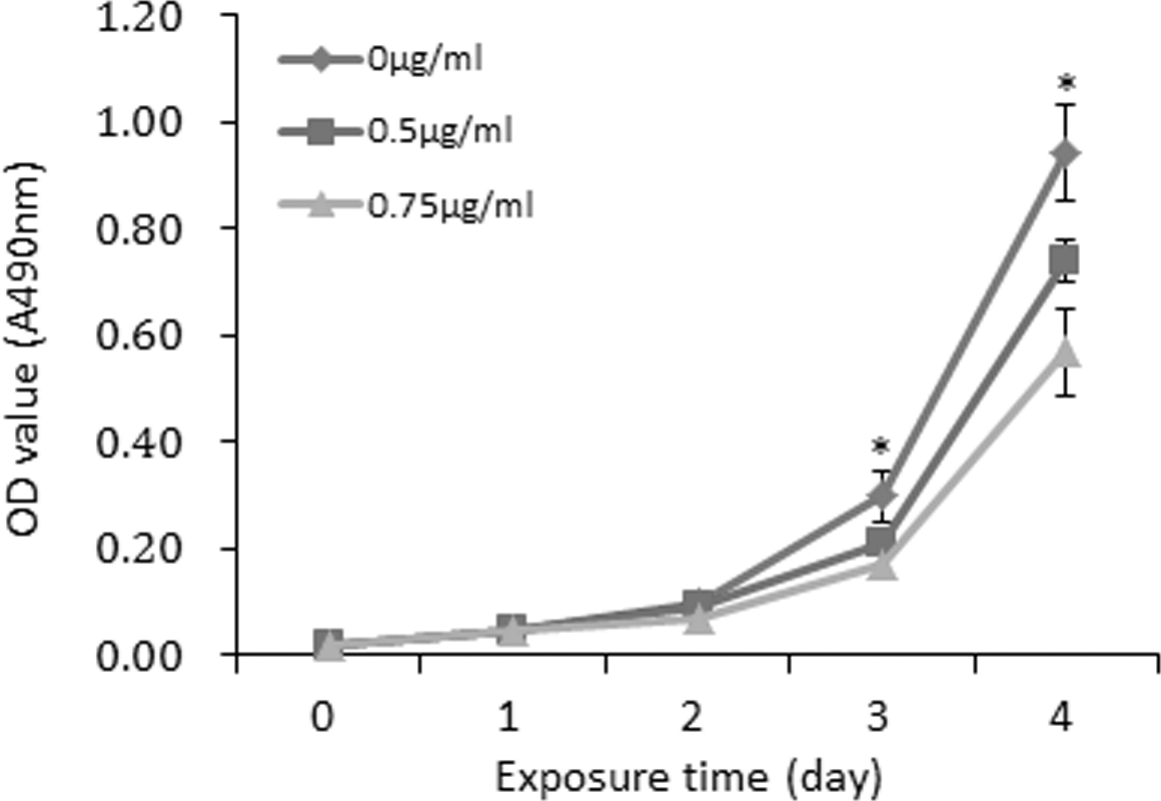
Recombinant FSTL1 protein retards the proliferation of CNE2 cells *in vitro* MTT assay of proliferation of CNE2 parental cells incubated with soluble recombinant FSTL1 protein at a concentration of 0 μg/ml, 0.5 μg/ml and 0.75 μg/ml, respectively. Data are mean ± SD of three independent experiments. *P < 0.05.

Because FSTL1 is a secreted protein, it may function both in the intracellular and extracellular environment. We analyzed FSTL1 level in serum and found no significant difference between NPC patients and healthy donors (Figure S1). We treated parental CNE2 cells with recombinant FSTL1 protein at 0.5 and 0.75 μg/ml. The data suggest that extracellular FSTL1 dose-dependently suppressed the proliferation of CNE2 cells (*P* < 0.05, Figure 6).

### Inactivation of FSTL1 might lead to loss of macrophage function in NPC by decreasing the expression of IL-1β and TNF-α

**Figure 7:**
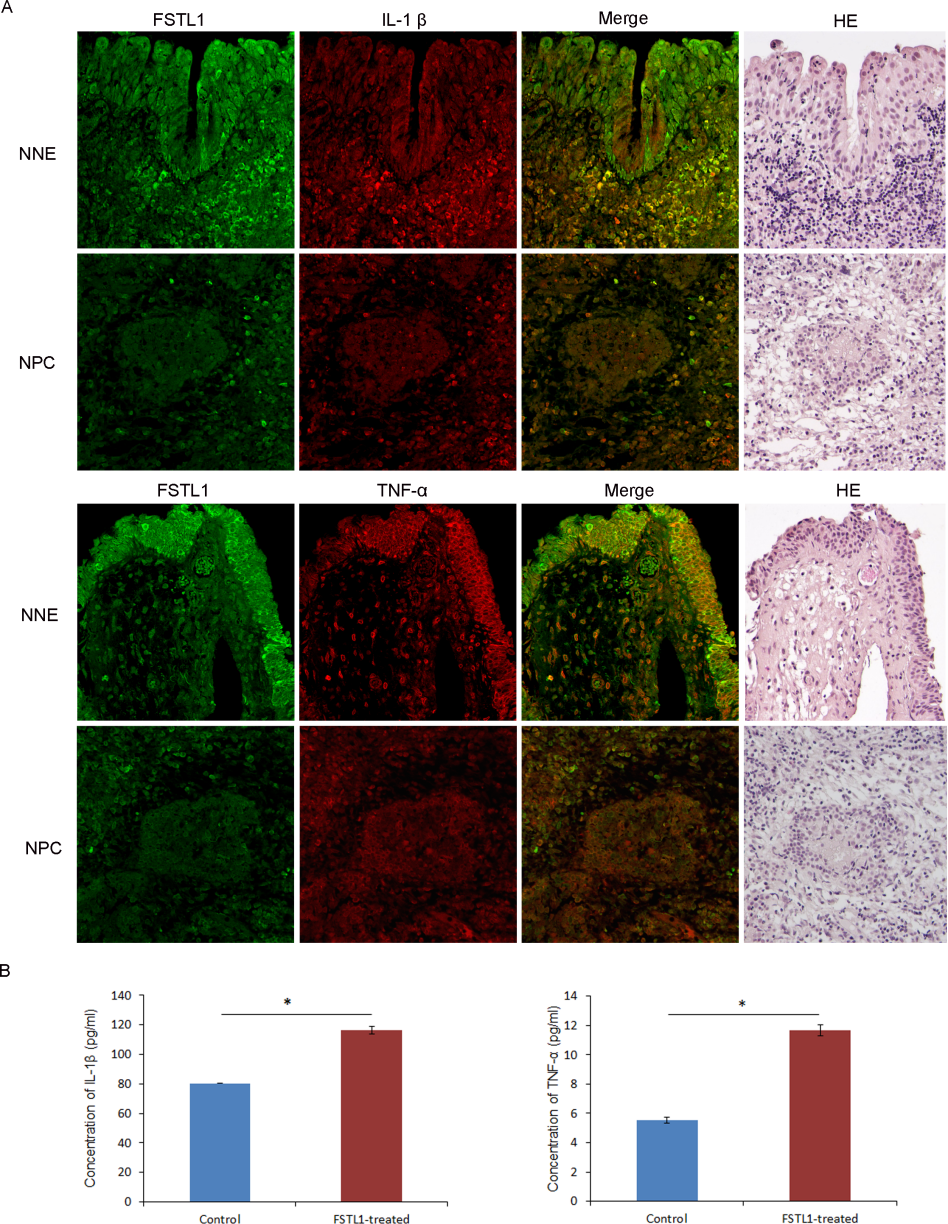
Decreased expression of FSTL1 associated with reduced IL-1β and TNF-α expression in NPC primary tumors (**A**) Human normal nasopharyngeal epithelium tissues (NNE) and NPC tumor tissues were double immunofluorescence stained with FSTL1 (green) and TNF-α (red) or IL-1β (red) antibodies. H & E staining demonstrates the histomorphological characteristic of sections. Magnification, 200×. (**B**) ELISA of IL-1β and TNF-α secretion after treatment of human macrophages with soluble FSTL1 (0.75 μg/ml). Mock treatment was a control. Data are mean ± SD (*n* = 3). *P < 0.05.

We hypothesized that the epigenetic downregulation of *FSTL1* we observed in NPC cell lines and biopsies might also result in reduced expression and secretion of proinflammatory cytokines in NPC or adjacent stroma. To verify this, we firstly demonstrated the inactivation of FSTL1 protein at NPC by immunohistochemistry staining (Figure S2), which confirmed our previous findings. Then, we performed double immunofluorescence staining of FSTL1 with IL-1β or FSTL1 with TNF-α in 15 primary NPC biopsies and 15 NNE samples. Again, these results revealed that FSTL1 strongly expressed in the cytoplasm of NNE tissue, with weaker signals in NPC tissue. And the expression of IL-1β and TNF-α was reduced in both NPC and adjacent stroma (Figure 7A), which indicates an immunosuppression state in NPC, as was previously suggested [18, 19].

### Recombinant soluble FSTL1 induces expression of IL-1β and TNF-α in cultured macrophages

Inspired by the possible colocalization of FSTL1 and IL-1β or FSLT1 and TNF-α at macrophages, we tried to investigate whether FSTL1 affects the secretion of IL-1β and TNF-α from macrophages. We generated macrophages from mononuclear cells isolated from a healthy donor, subsequently stimulated by rhM-CSF and verified by immunofluorescence staining with CD14 (data not shown). Then, we treated macrophages with recombinant human FSTL1 protein (0.75 μg/ml) for 7 days and tested the levels of IL-1β and TNF-α in cell culture supernatant. IL-1β and TNF-α secretion was significantly increased upon the treatment (*P* < 0.05, Figure 7B). Thus, epigenetic inactivation of *FSTL1* might be involved in the dysregulation of the immune system in NPC.

## DISCUSSION

A decreased expression of *FSTL1* in a panel of human cancers suggest a tumor-suppressive function for it. Indeed, overexpression of *FSTL1* suppressed the proliferation ability of lung cancer cells [14]. In ovarian cancer, restored *FSTL1* expression could inhibit tumor cell migration by reducing the secretion of matrix metalloproteinase 2 (MMP2). Exogenous *FSTL1* induced cell cycle arrest and cell apoptosis by activating important members of the cell apoptosis pathway, such as Fas cell surface death receptor (FAS), Fas ligand (FASLG), TNFRSF1A-associated via death domain (TRADD), caspase-3, caspase-7 and poly (ADP-ribose) polymerase (PARP) [15]. It was suggested involvement of Cx43 cellular factor to the FSTL1-mediated tumor suppression [20]. Moreover, it was demonstrated the post-transcriptional regulation of *FSTL1*. *FSTL1* was inactivated by miR-206 with the introduction of MyoD [21]. In line with these studies, our data demonstrated that *FSTL1* mRNA expression was downregulated in NPC cell lines and primary human tumor biopsies. Further, we determined that the inactivation of *FSTL1* was due to its promoter hypermethylation. Highly frequent promoter hypermethylation of *FSTL1* could be detected not only in advanced and also at early-stage NPC tumors, indicating that hypermethylation of *FSTL1* as one of initiating event in NPC tumorigenesis.

In order to reveal the role of *FSTL1* in NPC pathogenesis, we performed *in vitro* and *in vivo* studies. As a pro-inflammatory protein, FSTL1 was found to increase the synthesis of pro-inflammatory cytokines and chemokines by immune cells both *in vitro* and *in vivo*. FSTL1 was previously shown to induce the production of IL-1β, TNF-α, and IL-6 in macrophages and fibroblasts [16, 22] via Toll-like receptor 4/NF-κB signaling pathway [23, 24]. Another possible mechanism is that FSTL-1 activate the NLRP3 inflammasome in macrophages, leading to an increased secretion of IL-1β [25]. We therefore hypothesized that inhibition of FSTL1 might contribute to the escape of NPC tumor cells from immune surveillance. Here, we demonstrated epigenetic silencing of FSTL1 in NPC cell lines and biopsies. This could increase the tumorigenicity of NPC cells while simultaneously enhancing their capacity to escape recognition and elimination by innate immune cells such as stromal macrophages. In this study, we identified decreased FSTL1 expression in NPC tissue sections, concomitant with downregulation of IL-1β and TNF-α in tumor tissue macrophages. These findings suggest that epigenetic inactivation of FSTL1 short-cuts the paracrine secretion of FSTL1 by NPC cells, thus suppressing the inflammatory and anti-proliferative responses of innate immune cells in the cancer stroma.

The role of *FSTL1* in tumor invasion and metastasis is complex. In NPC cells, we found that ectopic expression of *FSTL1* inhibited tumor cell migration and invasion. However, one report in 2013 [26] showed that *FSTL1* plays a dual role in cancer bone metastasis, firstly by mediating tumor invasion and bone tropism, and secondly, by expanding the population of pluripotent mesenchymal stem-like cells. Our results appear to contradict this finding, but the discrepancy may be due to differences in histological origins of the malignancy. Interestingly, these authors also demonstrated that the expansion of mesenchymal stem-like cells was caused by inhibition of host antitumor immune responses mediated by *FSTL1* [26, 27], thus, this finding would be in agreement with our results.

Over the past decades, many tumor suppressor genes have been found to be inactivated by promoter hypermethylation in NPC, indicating that epigenetic silencing of tumor suppressor genes is one of the major molecular alterations in the process of NPC carcinogenesis. However, only a few of them are capable to serve as therapeutic targets. FSTL1 is a soluble secreted protein, treatment with recombinant FSTL1 protein inhibited proliferation of NPC cells, and induced host immune cell function as well. These make *FSTL1* an ideal therapeutic target, especially suitable to be developed as an anticancer drug. Further study is necessary to explore this potential.

## MATERIALS AND METHODS

### Ethics statement

Ethical permission for this study was granted by the Ethical Review Committee of the First Affiliated Hospital of Guangxi Medical University. Informed consent was obtained from all donors.

### Cell lines and sample collection

Six NPC cell lines (CNE1, CNE2, HONE1, HNE1, TW03 and C666-1) [28–30] were maintained at 37°C in IMDM medium (Invitrogen, Carlsbad, CA, USA) supplemented with 10% fetal calf serum (HyClone, UK Ltd, Northumberland, UK). Treatment with 5-aza-dC (Sigma, St. Louis, Mo., USA) was performed as described [31]. To obtain human monocyte-derived macrophages, peripheral blood mononuclear cells (PBMCs) were isolated from a healthy donor by use of a lymphocyte separation medium (Solarbio, Beijing, China) and cultured in appropriate cell culture medium in the presence of 1000 U/ml human macrophage-colony stimulating factor (rhM-CSF, PeproTech, Princeton, NJ, USA) for 7 days.

Primary NPC tumor biopsies were obtained from 70 newly diagnosed and untreated cases. The diagnoses were established by experienced pathologists according to the World Health Organization (WHO) classification. A total of 30 normal nasopharyngeal epithelial (NNE) samples obtained by tonsillectomy were used as controls. In all, 25 of the 60 NPC biopsies and 8 of the 20 NNE samples were processed for RNA extraction; the remaining 35 NPC and 12 NNE samples were used for DNA extraction. Another group of formalin-fixed and paraffin-embedded (FFPE) were used for immunofluorescence staining (15 NPC and 15 NNE samples)_and immunohistochemistry staining (10 NPC and 10 NNE samples).

### Semi-quantitative RT-PCR

Total RNA extraction, first-strand synthesis of cDNA and RT-PCR was performed as described [32]. The primer sequences were for *FSTL*1-RT-forward: CCA GACCACGATGTGGAAAC; *FSTL1*-RT-reverse: TTGCA TTGCTCAATGCA GAG; *GAPDH*-forward: AAGCTCAC TGGCATGGCCTT; and *GAPDH*-reverse: CTCTCTTCC TCTTGTGCTCTTG, generating a 186-bp (*FSTL1*) or 375-bp (*GAPDH*) amplification product. PCR programs were 94°C for 30 s, 58°C for 30 s (*FSTL1*) or 60°C for 30 s (*GAPDH*), then 72°C for 30 s and 30 cycles (*FSTL1*) or 24 cycles (*GAPDH*). The amplified PCR products were then visualized after electrophoresis in 2% agarose gel and semi-quantitative analysis involved use of Quantity One v4.4.0 (Bio-Rad Life Science, USA).

### DNA bisulfite treatment and promoter methylation analysis

DNA extraction, sodium bisulfite modification of DNA, methylation-specific PCR (MSP) and bisulfite genomic sequencing (BGS) were conducted as described [33, 34]. The primer sequences distinguishing unmethylated (U) and methylated (M) alleles were for *FSTL1*-M-forward: TCGAGGTTGGCGATCGGC; *FSTL1*-M-reverse: CGCAA ACTCGCTCCGACCG; *FSTL1*-U-forward: TTTTGAGG TTGGTGATTGGT; *FSTL1*-U-reverse: CACAAACTCA CTCCAACCA. Cycling conditions were initial denaturation at 95°C for 3 min, 40 cycles of 94°C for 30 s, 60°C (M) or 56°C (U) for 30 s, and 72°C for 30 s. MSP amplifications were performed in duplicate. To reveal the detailed methylation status of the promoter region, a set of BGS-PCR primers was designed for nucleotides −288 to −47 bp relative to the transcription start point of *FSTL1*. The primer sequences were as for *FSTL1*-BGS-forward: GGAAGGAGAGG TTTTAA and *FSTL1*-BGS-reverse: CTAACCTAAAAAACTTACT. BGS-PCR products were subcloned and transformed into JM109-competent cells. At least 5 clones for each sample were randomly selected and sequenced by using the cycle sequencing kit BigDye Terminator 3.0 (Applied Biosystems, Foster City, CA, USA) on an ABI 3100 sequencer.

### Vector construction and transfection

The full-length coding sequence for *FSTL1* from Origene (Rockville, MD, USA) was subcloned into the pCMV-Tag3A vector (Stratagene, La Jolla, CA, USA). The NPC cell line CNE2, which showed inactivation of *FSTL1*, was transfected with the pCMV-Tag3A-*FSTL1* plasmid (*FSTL1*-CNE2) or empty-vector pCMV-Tag3A plasmid (empty vector-CNE2) by using Lipofectamine 2000 (Invitrogen). Stable clones were obtained by G418 selection (400 μg/ml) for 2 weeks and maintained in medium containing 200 μg/ml G418.

### Cell proliferation assay

Stably transfected *FSTL1*-CNE2 and empty vector-CNE2 cells were seeded into 96-well plates at 2 × 10^3^ cells/well. Cell density was examined at different times by using the vital stain 3-(4, 5-dimethylthiazol-2-yl)-2, 5-diphenyltetrazolium bromide (MTT, Solarbio), and absorbance (OD490 nm) was measured by use of a iMark microplate reader (Bio-Rad, Hercules, CA, USA). MTT assay was performed to determine the effect of recombinant FSTL1 protein (Sino Biological Inc., Beijing, China) on proliferation of parental CNE2 cells.

### Colony-formation assay and wound healing assay

Wound healing assay and colony formation assay were performed as described [32].

### *In vitro* cell invasion assay

*In vitro* cell invasion assay involved the BD BioCoat Matrigel Invasion Chamber (BD Biosciences, Bedford, MA, USA). An amount of 5% fetal bovine serum was added to the cell culture medium in the lower chamber as a chemoattractant, and a cell suspension containing 2.5 × 10^4^ stably transfected cells was seeded immediately in the upper chamber. After incubation for 16 and 24 h, cells that had not invaded were removed from the upper chamber and cells that had invaded the lower surface of the membrane were fixed and stained with 1% crystal violet. Stained cells were photographed under a microscope.

### Flow cytometry

Flow cytometry was performed to determine the effect of *FSTL1* on cell apoptosis. An amount of 5 × 10^5^ transiently transfected cells was collected, washed twice with 1 × PBS, and resuspended in 500 μl of 1 × Binding buffer, followed by Annectin V/propidium iodide (PI) labeling in the dark (KeyGen BioTECH, Nanjing, China) for 15 min, then samples were analyzed by BD FACS Calibur with CellQuest software (San Jose, CA, USA).

### Western blot analysis

Western blot analysis was performed according to standard protocols. After protein blotting, membranes were incubated with primary antibodies for myc-tag (1:1000, Santa Cruz Biotechnology, Inc., CA, USA) and caspase-3 (1:1000, Cell Signaling Technology, Beverly, MA, USA) at 4°C overnight, then horseradish peroxidase-conjugated immunoglobulin G antibody (1:10000, Santa Cruz) at room temperature for 1 h. Antibody for GAPDH (1:1000, sc-32233, Santa Cruz) was an internal control.

### *In vivo* tumor formation assay

Eight female 6-week-old Balb/c athymic nude mice (Experimental Animal Center of Guangxi Medical University, China) were used. The experimental protocol was approved by the Ethical Review Committee of First Affiliated Hospital of Guangxi Medical University, and the committee's ethical guidelines for animal experimentation were followed. An amount of 2 × 10^6^ stably transfected *FSTL1*-CNE2 cells was injected subcutaneously into the right flank of nude mice. An equal amount of empty vector-CNE2 cells was injected into the left flank of mice as a control. The growth of tumors was monitored every 2 days for 2 weeks. Tumor volume (V) was calculated as V = (π/6) L × W × H, where L, W, and H represent tumor diameter in 3 mutually orthogonal planes. The animals were killed and tumors were excised and weighed on day 14.

### Double immunofluorescence staining and immunohistochemistry staining

Formalin-fixed and paraffin-embedded (FFPE) sections were deparaffinized and rehydrated before antigen retrieval and blocking. Sections were co-incubated with the antibodies for FSTL1 (1:200, Abcam, Cambridge, MA, UK) and IL-1β (1:200, Abcam) or FSTL1 and TNF-α (1:200, Abcam). As a secondary antibody-only control, PBS was used instead of primary antibodies. After overnight incubation at 4°C, slides were washed with PBS and incubated with secondary antibodies (1:400, Alexa Fluor 488 donkey anti-goat IgG, Molecular Probes, or Rhodamine 568-labeled anti-rabbit IgG, Chemicon International, Temecula, CA, USA) for 1 h at room temperature. Finally, nuclei were counterstained with 4′-6-diamidino-2-phenylindole (DAPI) and sections were examined under a confocal laser scanning microscope (Fluoview FV1000-D, Olympus, Tokyo, Japan). H & E staining for histology analysis was performed as described [35]. Immunohistochemistry staining was done as we described before [36].

### Enzyme-linked immunosorbent assay (ELISA)

In brief, *in vitro*-derived macrophages were cultured with 0.75 μg/ml recombinant human FSTL1 protein at 37°C with 5% CO_2_ for 7 days. Secretion of TNF-α and IL-1β in the cell culture supernatant were determined by ELISA assay (Cusabio Biotech, Newark, DE, USA) and quantified by comparing the OD450 nm value of samples to the standard curve. The experiment was performed in triplicate. The concentration of FSTL1 protein in serum were detected by ELISA assay (Cusabio Biotech, Newark, DE, USA) according to the instruction.

### Statistical analysis

SPSS v18.0 (SPSS Inc., Chicago, IL, USA) was used for statistical analysis. The Pearson chi-square test or Fisher's exact test was used to analyze the association of clinical pathological features of NPC patients and methylation status of *FSTL1*. Student *t* test was used to compare two independent groups, while One-way ANOVA was used to compared means of three independent groups. *P* < 0.05 was considered statistically significant.

## SUPPLEMENTARY MATERIALS FIGURES


