# Supplementary material for: Epigenetic inactivation of follistatin-like 1 mediates tumor immune evasion in nasopharyngeal carcinoma
Source: Oncotarget. 2016 Feb 24;7(13):16433–44. doi: 10.18632/oncotarget.7654 (PMC4941326; doi:10.18632/oncotarget.7654)
Supplement: Supplementary file 1 [file oncotarget-07-16433-s001.pdf]

Epigenetic inactivation of follistatin-like 1 mediates tumor immune evasion in nasopharyngeal carcinoma

Supplementary Materials

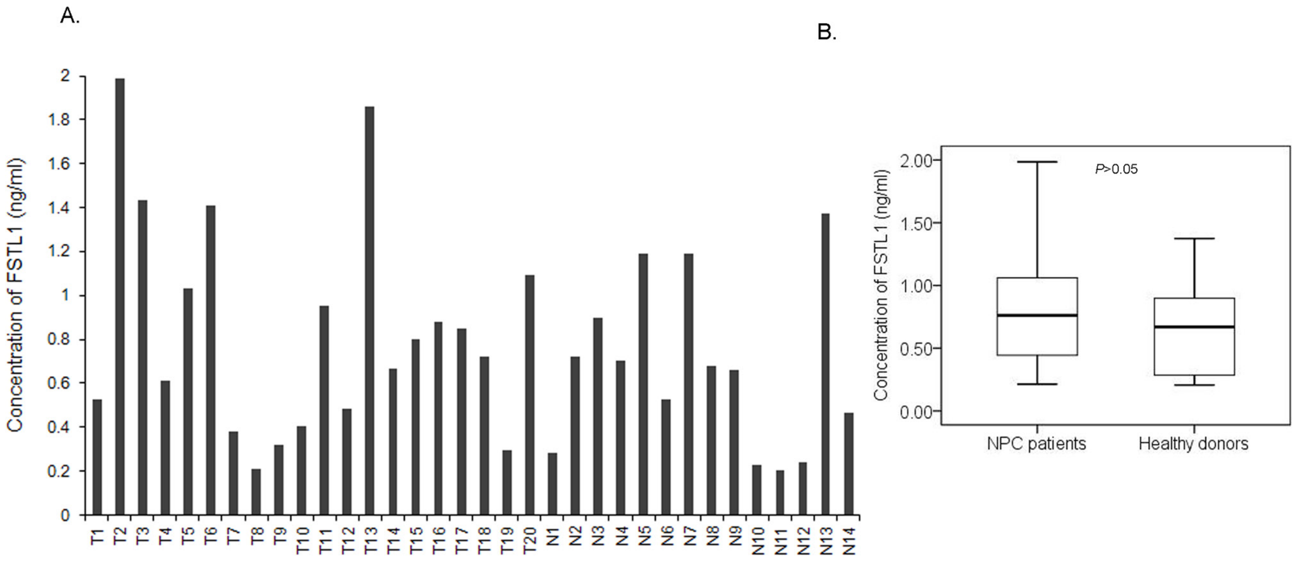

Supplementary Figure S1: Determine the concentration of FSTL1 in serum by ELISA assay. (**A**) Bar graph shows FSTL1 protein level in the serum of NPC patients (n = 20) and healthy donors (n = 14). (**B**) The box plots summarize the concentration of FSTL1 in serum. Boxes indicate 25 to 75 percentile, horizontal line indicates the mean, and bars indicate 10 and 90 percentile.

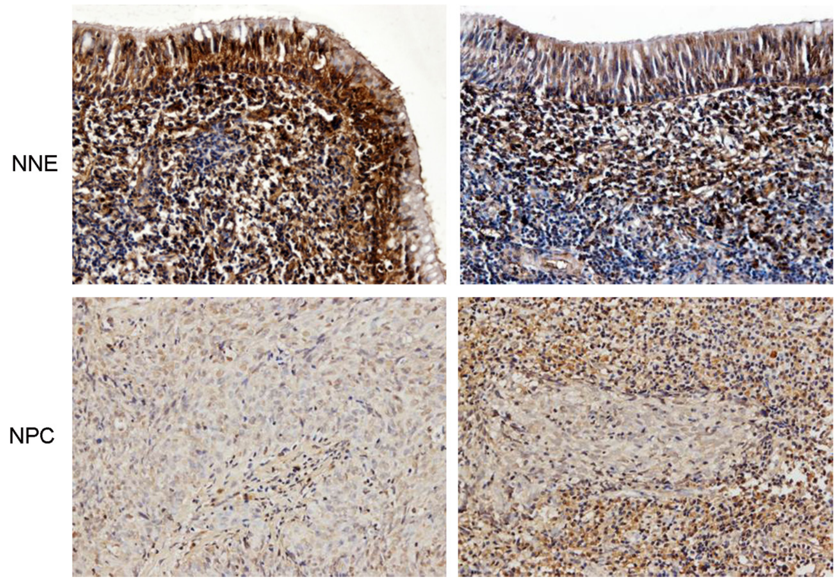

Supplementary Figure S2: Detection of FSTL1 expression in NPC biopsies and NNE samples by immunohistochemistry staining. Representative slides of NPC and NNE samples stained by anti-FSTL1 antibody.
